# Intratumoral administration of astatine-211-labeled gold nanoparticle for alpha therapy

**DOI:** 10.1186/s12951-021-00963-9

**Published:** 2021-07-28

**Authors:** Hiroki Kato, Xuhao Huang, Yuichiro Kadonaga, Daisuke Katayama, Kazuhiro Ooe, Atsushi Shimoyama, Kazuya Kabayama, Atsushi Toyoshima, Atsushi Shinohara, Jun Hatazawa, Koichi Fukase

**Affiliations:** 1 Department of Nuclear Medicine and Tracer Kinetics, Osaka University Graduate School of Medicine, 2-2 Yamadaoka, Suita, Osaka 565-0871 Japan; 2 Department of Chemistry, Graduate School of Science, Osaka University, 1-1 Machikaneyama, Toyonaka, Osaka 560-0043 Japan; 3 Division of Science, Institute for Radiation Sciences, Osaka University, 1-1 Machikaneyama, Toyonaka, Osaka 560-0043 Japan; 4 Research Center for Nuclear Physics, Osaka University, 10-1 Mihogaoka, Ibaraki, Osaka 567-0047 Japan

**Keywords:** Astatine-211, Alpha emitters, Gold nanoparticles, Radiolabeling, Cancer therapy

## Abstract

**Background:**

^211^At is a high-energy α-ray emitter with a relatively short half-life and a high cytotoxicity for cancer cells. Its dispersion can be imaged using clinical scanners, and it can be produced in cyclotrons without the use of nuclear fuel material. This study investigated the biodistribution and the antitumor effect of ^211^At-labeled gold nanoparticles (^211^At-AuNP) administered intratumorally.

**Results:**

AuNP with a diameter of 5, 13, 30, or 120 nm that had been modified with poly (ethylene glycol) methyl ether (mPEG) thiol and labeled with ^211^At (^211^At-AuNP-S-mPEG) were incubated with tumor cells, or intratumorally administered to C6 glioma or PANC-1 pancreatic cancers subcutaneously transplanted into rodent models. Systemic and intratumoral distributions of the particles in the rodents were then evaluated using scintigraphy and autoradiography, and the changes in tumor volumes were followed for about 40 days. ^211^At-AuNP-S-mPEG was cytotoxic when it was internalized by the tumor cells. After intratumoral administration, ^211^At-AuNP-S-mPEG became localized in the tumor and did not spread to systemic organs during a time period equivalent to 6 half-lives of ^211^At. Tumor growth was strongly suppressed for both C6 and PANC-1 by ^211^At-AuNP-S-mPEG. In the C6 glioma model, the strongest antitumor effect was observed in the group treated with ^211^At-AuNP-S-mPEG with a diameter of 5 nm.

**Conclusions:**

The intratumoral single administration of a simple nanoparticle, ^211^At-AuNP-S-mPEG, was shown to suppress the growth of tumor tissue strongly in a particle size-dependent manner without radiation exposure to other organs caused by systemic spread of the radionuclide.

**Graphic Abstract:**

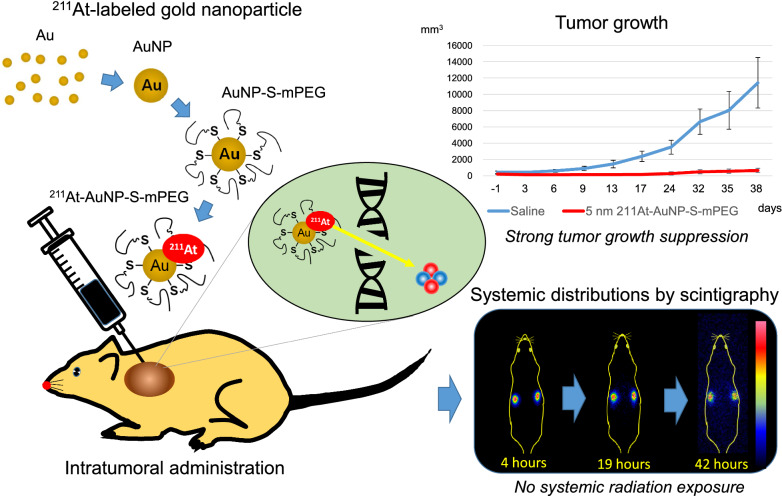

**Supplementary Information:**

The online version contains supplementary material available at 10.1186/s12951-021-00963-9.

## Background

As a local radiation therapy for cancer and in addition to external irradiation with γ-rays or X-rays, the effectiveness of brachytherapy using a sealed X-ray source for prostate cancer [1], breast cancer [2, 3], uterine cancer [4, 5], head and neck cancer [6, 7], and brain tumors [8], is well known. However, the invasiveness of the procedure, extra-lesion displacement of the sealed radiation source, or adverse effects caused by the leakage of X-rays from radionuclides with a long half-life into adjacent organs are problematic issues. Adverse effects on other organs and psychological adverse effects of brachytherapy with γ-rays and X-rays have also been reported [9]. Brachytherapy for breast cancer is prone to causing skin ulcers, infections, rib fractures, and local pain, and its therapeutic effect is equivalent [10] or inferior [11] to external beam radiation therapy (EBRT). As for brachytherapy for brain tumors [12–14], although the therapeutic effect has been better than ERBT in some cases [14] or about the same [13], adverse effects such as vomiting, headaches and cerebral edema are relatively common [8].

β-Ray-emitting or α-ray-emitting nuclides have a high linear energy transfer (LET) and relative biological effectiveness and are particularly toxic to proliferating cells. Because of the short range of β-ray or α-ray emitters, normal tissues are minimally exposed if the radiation sources are appropriately distributed. This property can overcome the adverse effects of conventional EBRT and brachytherapy caused by γ-rays or X-rays. Thus, brachytherapy using high-LET radiation is feasible using locally distributed small seeds labeled with β- or α-ray emitting nuclides. Yook et al. showed that the β-ray-emitting nuclide ^177^Lu could be used to label gold nanoparticles (AuNP) that could then be locally administered to breast cancer xenografts to suppress tumor growth [15]. Zhu et al. clarified that poly(cyclotriphosphazeneco-polyethylenimine) nanospheres labeled with radionuclide ^131^I had anticancer capabilities when injected intratumorally [16]. Salvanou et al. demonstrated that α-ray-emitting nuclide ^225^Ac-labeled AuNP could be used to inhibit tumor growth when administered intratumorally to glioma xenografts [17]. However, these studies also showed that radionuclides could spread systemically to other organs after intratumoral administration at time points that were much earlier than the half-lives of the radionuclides. Systemic exposures to long half-life nuclides, such as ^177^Lu, ^131^I or ^225^Ac, are detrimental and should be minimized whenever radionuclides are administered intratumorally. Assuming that the radionuclides are not scattered externally from the tumor, extremely high doses of radioactivity could be administered repeatedly without exposing normal organs and tissues to radiation. On the other hand, since their ranges are extremely short, α-ray emitters release energy only within their short distribution ranges. Thus, controlling the tissue distribution after administration is pivotal, and might be possible by adjusting the seed size and performing additional seed modifications.

^211^At, which can be produced in cyclotrons without the use of nuclear fuel material, emits high-energy α-rays with a relatively short half-life and can be clearly imaged by clinical scanners or autoradiography. ^211^At has been recently labeled with various molecules including amino acid transporter substrates, inhibitors of prostate-specific membrane antigens, or antibodies against cancer-specific antigens for targeted alpha therapy of cancer via intravenous administration [18–20]. On the other hand, as far as we know, no treatments for antineoplastic tumors with ^211^At-labeled AuNP have been reported so far. The objective of this study was to propose an effective nanoseed brachytherapy with significantly reduced radiation exposure. In the present study, we investigated the systemic and intratumoral distributions and verified the antitumor effect of ^211^At-labeled AuNP administered intratumorally.

## Methods

### Animal models

Male nude rats (F344/NJcl-rnu/rnu; 7 weeks old) were purchased from CLEA Japan, Inc. (Tokyo, Japan), and male nude mice (BALB/cSlc-nu/nu) were purchased from Japan SLC Inc. (Tokyo, Japan).

C6 glioma cells, which are a rat cell line derived from a glial cell tumor induced by N-nitrosomethylurea, were used as tumor cells. C6 glioma cells are suitable for assessing growth inhibition and the tissue retention of nanoparticles against wash-out in blood flow because they generally have a high growth rate and a high level of vascularization. C6 glioma cells obtained from RIKEN BRC were cultured using modified Eagle medium (MEM) (Sigma-Aldrich Japan, Tokyo, Japan) supplemented with 10% fetal bovine serum in a humidified incubator at 37 °C under 5% CO_2_.

Human pancreatic cancer cells (PANC-1) were obtained from the American Type Culture Collection. The cells were cultured in RPMI1640 medium with L-glutamine and phenol red (Fujifilm Wako Pure Chemical, Tokyo, Japan) with 10% heat-inactivated fetal bovine serum and 1% penicillin–streptomycin.

C6 glioma cells (0.9 × 10^7^ cells/50 μL MEM) supplemented with 50 μL matrigel (Corning, New York, USA) were subcutaneously transplanted into both shoulders of the rats under anesthesia with 2.0% isoflurane in oxygen. PANC-1 cells (1.0 × 10^7^ cells/50 μL RPMI1640) supplemented with 50 μL matrigel were transplanted into the left shoulders of the mice by subcutaneous injection. A matrigel scaffold was used to create tumors of a certain size reliably, since such scaffolds can retain transplanted cells locally, promoting tumor engraftment and growth.

### In vitro* cell toxicity study*

The C6 rat glioma cell line and the PANC-1 cell line were cultured in Dulbecco’s MEM medium (FUJIFILM Wako Pure Chemical Corporation, Osaka, Japan) containing 10% fetal bovine serum (FBS) (Gibco™, Life Technologies, Carlsbad, CA USA) and 1% penicillin–Streptomycin Solution (FUJIFILM Wako Pure Chemical Corporation, Osaka, Japan). The C6 rat glioma cells (2 × 10^4^ cells/well in 100 µL medium) and PANC-1 cells (1 × 10^4^ cells/well in 100 µL medium) were then seeded onto 96-well plates and cultured for 1 day. Different sizes of AuNP modified with poly (ethylene glycol) methyl ether (mPEG) thiol (AuNP-S-mPEG) and ^211^At-AuNP-S-mPEG were synthesized (see Additional file 1) [21] and serially diluted; samples were then added to each well (25 µL/well). The mass concentrations of AuNP-S-mPEG are shown in Additional file 1: Table S3. After 24 h of incubation, we measured cell viability using a Cell Counting Kit 8 (CCK8) (Dojindo Laboratories, Kumamoto, Japan) and a microplate reader at 450 nm.

### Evaluation of intracellular uptake of AuNP-S-mPEG

C6 rat glioma cells were seeded onto 35 mm glass bottom dishes (4 × 10^5^ cells/dish in 2 mL medium) and cultured for 1 day. Different kinds of AuNP-S-mPEG nanoparticles were added to the dish, and the cultures were incubated for 24 h. After incubation, Hoechst solution was added to the dish, and the cultures were incubated for 10 min while being protected from light. The cells were washed 3 times with PBS, and 4% paraformaldehyde phosphate buffer solution was added to fix the samples for 30 min. After fixation, the samples were washed 3 times with PBS.

The cellular uptake of AuNP-S-mPEG was investigated using reflectance imaging from the cells [22]. Images were taken using a Nikon A1R + inverted confocal microscope (Nikon Corp., Tokyo, Japan) and a Plan Apo VC water-immersion objective lens (60 × , NA = 1.20; Nikon, Tokyo, Japan).

### Administration of AuNP-S-mPEG

Nanoparticles were intratumorally administered to 12 rats (24 tumors) 13 days after C6 glioma cell transplantation and to 12 mice (12 tumors) 14 days after PANC-1 cell inoculation under anesthesia with 2.0% isoflurane in oxygen. Solutions of saline (for 3 rats, 6 tumors), AuNP-S-mPEG + saline (for 3 rats, 6 tumors), 120 nm ^211^At-AuNP-S-mPEG + saline (for 4 rats, 8 tumors), 30 nm ^211^At-AuNP-S-mPEG + saline (for 4 rats, 8 tumors), 13 nm ^211^At-AuNP-S-mPEG + saline (for 3 rats, 6 tumors, and for 6 mice, 6 tumors), and 5 nm ^211^At-AuNP-S-mPEG + saline (for 3 rats, 6 tumors) were prepared in a manner such that the solution volume was equal to the corresponding tumor volume. Solutions of saline (for 3 rats, 6 tumors), unlabeled 30 nm AuNP-S-mPEG + saline (for 3 rats, 6 tumors), and unlabeled 13 nm AuNP-S-mPEG + saline (for 6 mice, 6 tumors) were also prepared in the same way for use in the control subjects.

Each of the solutions was slowly administered into the tumor over a time period of about 1 min. For administration, the linear probe of an ultrasonic device (ProSound α6; Hitachi-Aloka Medical, Ltd., Tokyo, Japan) was used so that the needle tip of the syringe (Myjector 29G; Terumo Co. Ltd., Tokyo, Japan) was placed in the center of the tumors. The radioactivity administered per tumor was 1.4 ± 0.4 MBq for rats and 1.2 ± 0.1 MBq for mice (Additional file 1: Table S4).

### Follow-up and evaluation after administration

After intratumoral administration, the animals were monitored for 38 or 39 days; body weight and tumor size were measured under anesthesia using 2.0% isoflurane in oxygen. The major and minor diameters of each tumor were measured with a caliper, and the volume was determined by assuming that the tumor was a spheroid. Forty days after administration, the tumor was excised and the tumor mass was measured. After the experiments, the animals were euthanized with an excessive dose of isoflurane.

### Scintigraphy

Whole-body scintigraphy was performed at 4 and 19 h after the administration of ^211^At-AuNP-S-mPEG to rats; scintigraphy was performed using a gamma camera (E.cam; Siemens Healthcare, Erlangen, Germany) equipped with a low-energy, high-resolution and parallel-hole collimator. The detectors were set at a position 5 cm from the subject. Each animal was immobilized in a prone position under anesthesia with 2.0% isoflurane in oxygen, and imaging was performed for 10 min at 4 and 19 h after the administration of ^211^At to obtain planar images of the front and rear surfaces with a 256 × 256 matrix size (pixel size: 1.2 mm). For the 5 nm and 13 nm ^211^At-AuNP-S-mPEG groups, image acquisitions were also performed at 42 h after the administration of ^211^At-AuNP-S-mPEG.

### Autoradiography

All the tumors were excised from the rats on the day after the administration of ^211^At-AuNP-S-mPEG with a diameter of 120 nm (n = 2) or 30 nm (n = 2). The tumor specimens were then immediately frozen at -80 °C and sliced to a thickness of about 30 μm using a cryostat (CryoStar NX70; Thermo Scientific Inc., MA, USA), then attached to slide glasses. The frozen sections were then immediately dried with a dryer and placed in contact with an imaging plate for about 1 h. Imaging was performed using a nonconfocal variable mode laser scanner (Typhoon FLA 7000; GE Healthcare Life Sciences, Buckinghamshire, England). After the tumors were resected, the rats were euthanized by the administration of an excessive amount of isoflurane.

### Statistics

Statistical calculations were performed using SPSS 17.0. To compare tumor masses, a one-way ANOVA and the Levene test were performed, followed by the Tukey honestly significant difference (HSD) post-hoc test.

## Results

### Compound characterizations

Multi-angle dynamic light scattering (MADLS) and zeta-potential measurements and TEM imaging revealed the properties and stability of the AuNP particles of all sizes. Please see the text, Additional file 1: Tables S1–S3, and Fig. S1.

### In vitro* cytotoxicity and cellular internalization study*

C6 glioma cells were cultured with unlabeled 5 nm, 13 nm, 30 nm, or 120 nm AuNP-S-mPEG for 24 h, but no obvious effect on viability was observed even at a high concentration. AuNP-S-mPEG was thus determined to be non-cytotoxic regardless of the concentration or particle size (Additional file 1: Fig. S2A–D).

**Fig. 1:**
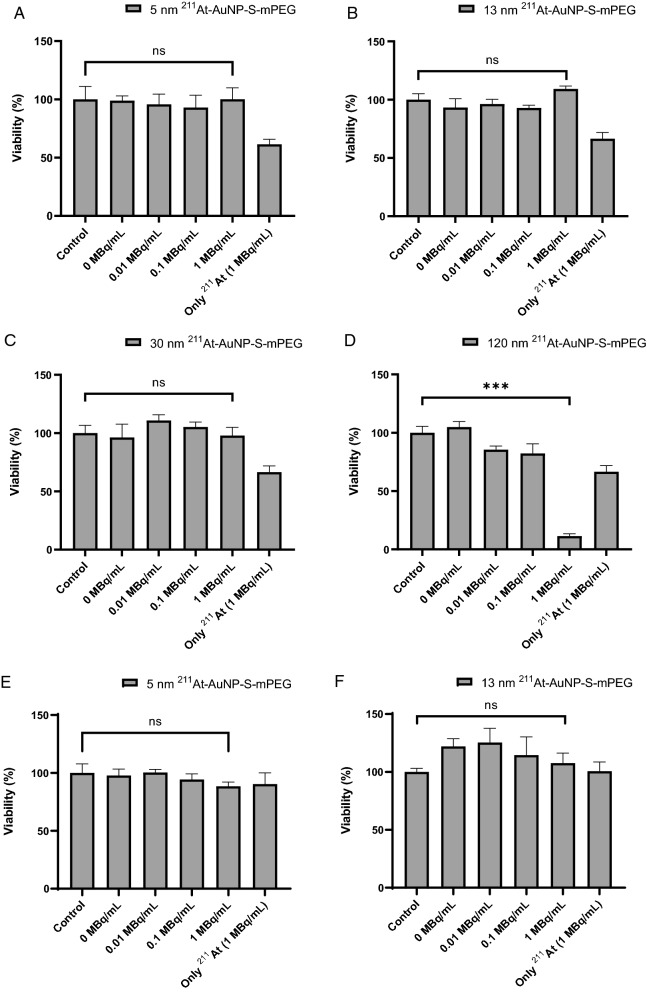

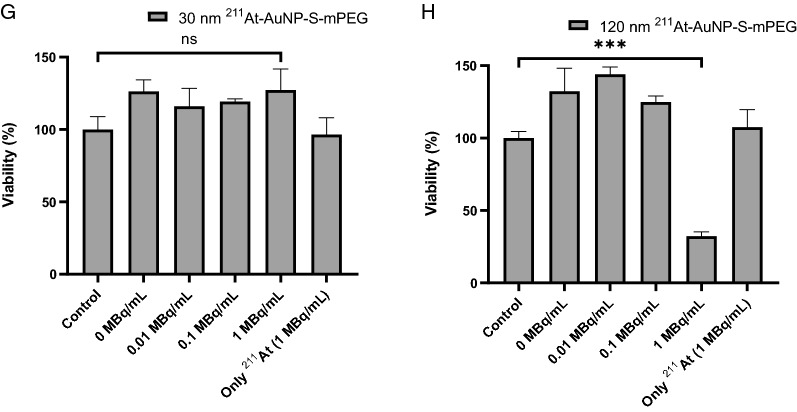
C6 glioma cells (**A**, **B**, **C**, **D**) and PANC-1 cells (**E**, **F**, **G**, **H**) were cultured for 24 h with saline as a control, 1 MBq/mL of ^211^At, or ^211^At-AuNP-S-mPEG labeled with 0 to 1 MBq/mL of radioactivity and a diameter of 5 nm (**A**, **E**), 13 nm (**B**, **F**), 30 nm (**C**, **G**) or 120 nm (**D**, **H**). For the evaluation of cell viability, CCK8 Kit was used. WTS-8 solution was added and the cells were cultured for 2 h. Viability was calculated by measuring the absorbance as shown in the formula below: $\mathrm{Viability}\left(\mathrm{\%}\right)=\frac{T-{T}_{0}}{C-{C}_{0}}$, where T is absorbance of the test cells, T_0_ is background of the test cells, C is the absorbance of the control cells, and C_0_ is the background of the control cells. The mass concentrations of AuNP-S-mPEG labeled with 1 MBq/mL ^211^At in (**A**, **B**, **C**, **D**) were almost the same level as those in (**A**, **C**, **E**, **G**) in Fig. 2, respectively (see Additional file 1: Table S3).

A significant decrease in viability was observed only in C6 glioma cells and PANC-1 cells cultured with 1 MBq/mL of 120 nm ^211^At-AuNP-S-mPEG (Fig. 1A–H). The mass concentrations of ^211^At-AuNP-S-mPEG in the treated cell cultures are shown in Additional file 1: Table S3.

**Fig. 2:**
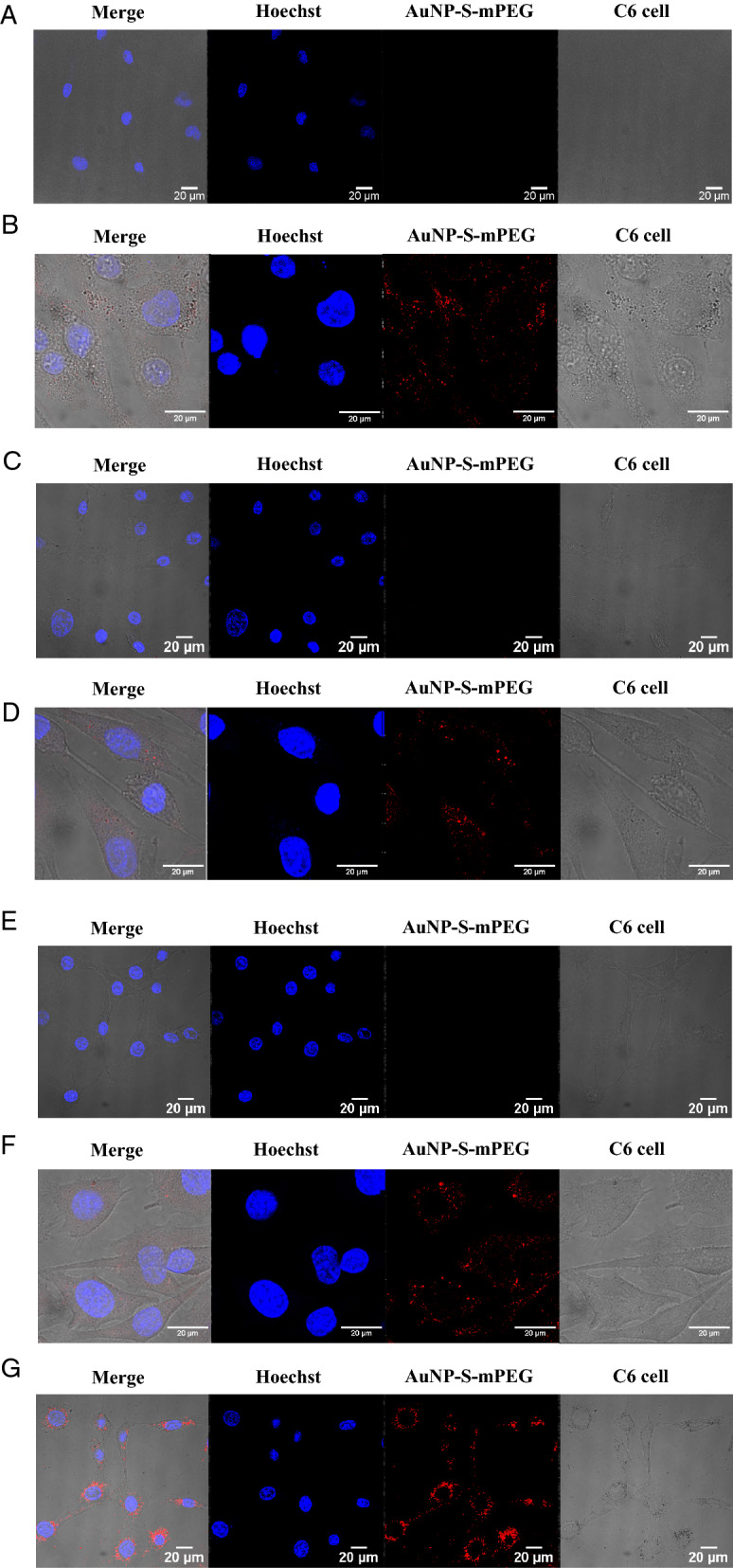
C6 glioma cells were seeded onto 35-mm glass bottom dishes and the cells were incubated with 5 nm, 13 nm, 30 nm or 120 nm AuNP-S-mPEG for 24 h. The intracellular uptake of these nanoparticles was examined using reflectance imaging. In each image, the leftmost section shows the bright field image of the cells, the second section shows the reflectance image of the particles taken up by the cells, the third section shows the fluorescent Hoechst staining for DNA, and the rightmost section shows the fused image. The C6 glioma cells were incubated with AuNP-S-mPEG with the following sizes and mass concentrations: (**A**) 5 nm and 12.7 mg/L, (**B**) 5 nm and 138.4 mg/L, (**C**) 13 nm and 6.9 mg/L, (**D**) 13 nm and 68.6 mg/L, (**E**) 30 nm and 8.7 mg/L, (**F**) 30 nm and 51.6 mg/L, and (**G**) 120 nm and 33.6 mg/L. The mass concentrations in (**A**), (**C**), (**E**), and (**G**) were almost the same as those of 1-MBq ^211^At-AuNP-S-mPEG in the cytotoxicity studies (**A**), (**B**), (**C**), and (**D**) illustrated in Fig. 1, respectively (see Additional file 1: Table S3)

When C6 glioma cells were cultured in the presence of AuNP-S-mPEG for 24 h, the 5 nm, 13 nm, 30 nm, and 120 nm AuNP-S-mPEG were internalized into the cells in a concentration-dependent manner (Fig. 2). The mass concentrations of AuNP-S-mPEG in the treated cell cultures are mentioned in the legend for Fig. 2. For the internalization of the 5 nm, 13 nm and 30 nm AuNP-S-mPEG, however, a concentration higher than that required by the 120 nm AuNP-S-mPEG was necessary. Precipitation, which was thought to have increased the concentration at the bottom of the culture significantly, was only observed in the 120 nm AuNP-S-mPEG group (Additional file 1: Fig. S3).

### Scintigraphy

**Fig. 3:**
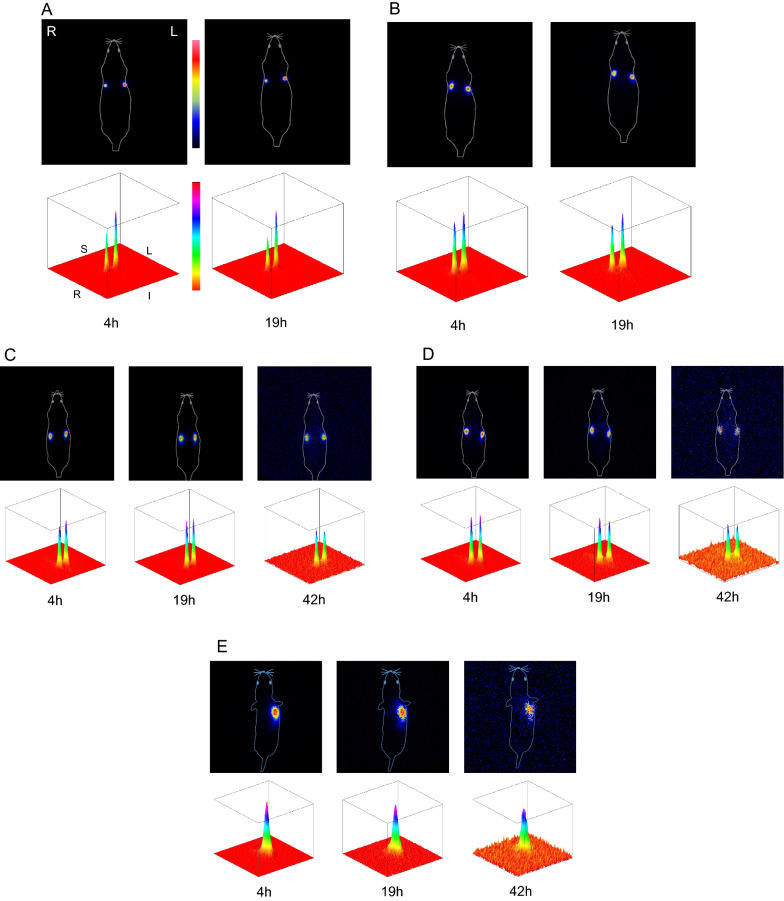
Scintigraphy of C6 glioma-bearing rats was performed at 4 and 19 h after the administration of 120 nm (**A**), 30 nm (**B**), 13 nm (**C**), or 5 nm (**D**) ^211^At-AuNP-S-mPEG. For the 13 nm (**C**) and 5 nm (**D**) ^211^At-AuNP-S-mPEG, imaging was also performed at 42 h after administration. Scintigraphy of PANC-1 bearing mice was also performed in a same way as rats at 4, 19, and 42 h after the administration of 13 nm ^211^At-AuNP-S-mPEG (E). The radioactivity distributions (upper rows) are also shown using surface plots (lower rows) that display a square field of view of the dorsal detector with side length 30 cm for rats (**A**–**D**), or 15 cm for mice (**E**), which covers the whole body of an animal. No systemic accumulation of radioactivity was observed in any of the organs. S: Superior, I: inferior, R: right, L: left

No ^211^At distribution was detected in any region other than the C6 tumor for all sizes of ^211^At-AuNP-S-mPEG at 4 and 19 h after administration (Fig. 3A-D). In the 5 nm and 13 nm ^211^At-AuNP-S-mPEG groups, no systemic ^211^At distribution was detected even at 42 h after administration (Fig. 3C, D). The 120 nm ^211^At-AuNP-S-mPEG were found to accumulate in spots within the tumor tissue, whereas the 5 nm, 13 nm, and 30 nm ^211^At-AuNP-S-mPEG diffused over a wider range than that observed for the 120 nm ^211^At-AuNP-S-mPEG. For PANC-1 pancreatic cancer as well as C6 glioma, ^211^At accumulated exclusively within the tumor after the administration of 13-nm ^211^At-AuNP-S-mPEG (Fig. 3E).

### Autoradiography of tumors

**Fig. 4:**
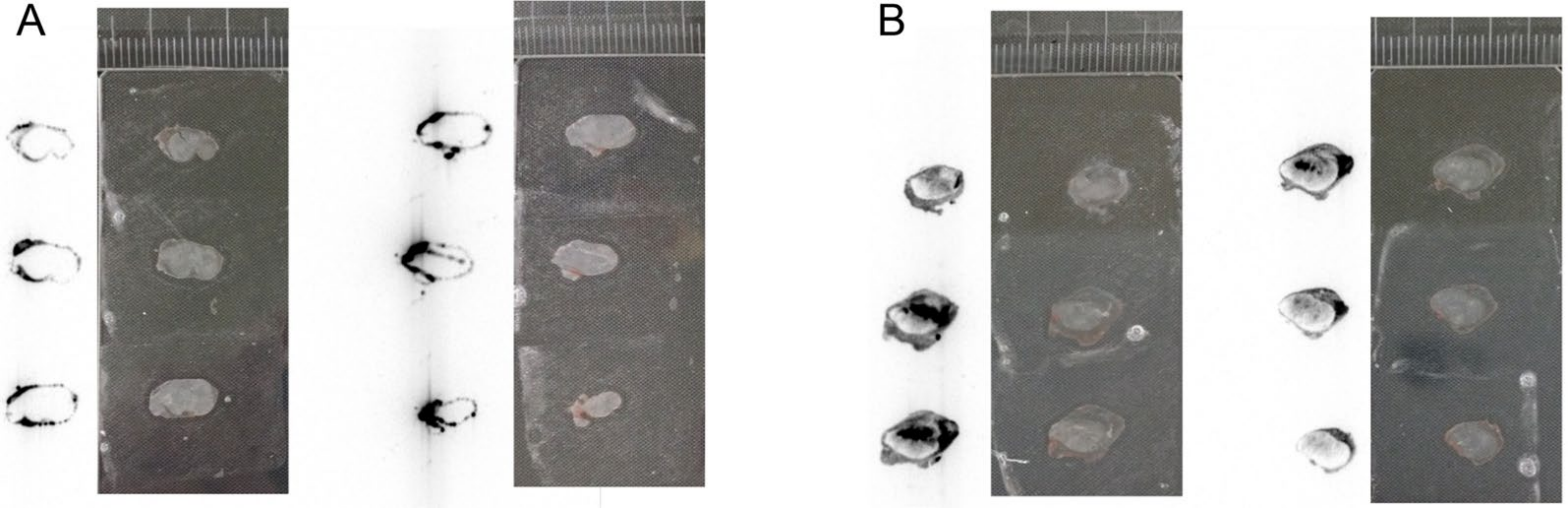
Twenty-four hours after the administration of 120 nm (**A**) and 30 nm (**B**) ^211^At-AuNP-S-mPEG, the tumors were excised, sliced and attached to a slide glass (right). Autoradiography (left) was performed after placing both tumor sections in contact with the same imaging plate for an hour

The ^211^At was observed in tumors excised from rats injected with 120 nm or 30 nm ^211^At-AuNP-S-mPEG. The distribution of the 120 nm ^211^At-AuNP-S-mPEG was confirmed to be remarkably uneven (Fig. 4A), whereas the 30 nm ^211^At-AuNP-S-mPEG were distributed almost throughout the entire tumor (Fig. 4B).

### Study of treatment efficacy

**Fig. 5:**
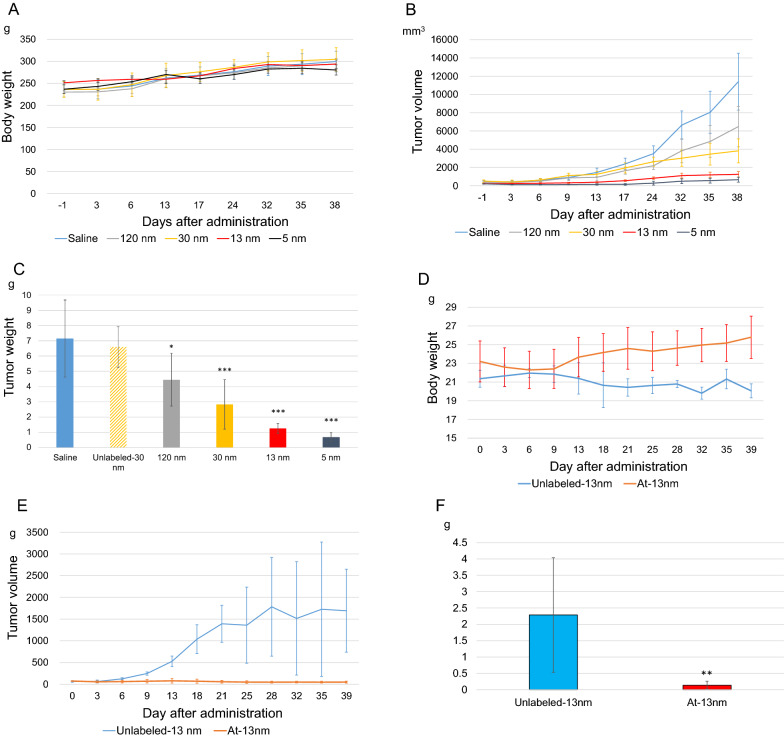
Changes in the body weights of the rats (**A**) and mice (D) and changes in the tumor volumes of the C6 glial cells (**B**) and PANC-1 cells (**E**) after intratumoral administration (Tumor size at the time of administration and the ^211^At dose were 334 ± 152 mm^3^ and 1.4 ± 0.4 MBq/tumor for rats and 72 ± 11 mm^3^ and 1.2 ± 0.1 MBq/tumor for mice, respectively). The C6 glial (**C**) and PANC-1 (**F**) tumors were excised 40 days after administration and their masses were measured. The error bars indicate the standard deviation. The tumor mass after the administration of ^211^At was significantly smaller than that seen in the controls. In PANC-1 xenografts, necrosis inside the tumor increased the size variability, leading to an increased standard deviation. The body weights of the mice in the control group were significantly lower than those in the group treated with ^211^At (*0.01 ≤ *p* < 0.05, **0.001 ≤ *p* < 0.01, ***p < 0.001)

All the animals survived for 38 days after administration. No abnormal changes in body weight were seen in any of the rats, and no significant differences in body weight were seen among the groups (Fig. 5A). On the other hand, the body weight in the control mice treated with unlabeled AuNP-S-mPEG was significantly lower than that in the mice treated with ^211^At-AuNP-S-mPEG. The C6 gliomas treated with the 5 nm ^211^At-AuNP-S-mPEG had the lowest growth rate, based on tumor size (Fig. 5B). Regarding the mass of the excised tumors, the group treated with the 5 nm ^211^At-AuNP-S-mPEG had the smallest tumor masses, which were significantly smaller than those of the control group. The mass of the excised tumor was proportional to the size of the ^211^At-AuNP-S-mPEG that had been used (Fig. 5C). Unlabeled AuNP-S-mPEG had no effect on tumor size.

Significant treatment effects were also confirmed in the PANC-1 xenograft models (Fig. 5D-F).

## Discussion

Consistent with the finding that the binding between AuNP and ^211^At is tight and stable [23], the labeling rate of ^211^At-AuNP-S-mPEG did not appear to change in the present study even after a time period equivalent to 6 half-lives of ^211^At. The mPEG modification was stable in saline, and AuNP-S-mPEG did not aggregate over 24 h (Additional file 1: Fig. S1 J-M). An in vitro experiment evaluating cytotoxicity against tumor cells showed that unlabeled AuNP-S-mPEG at nanoparticle sizes of 5 to 120 nm did not affect cell viability even after the cellular internalization of the nanoparticles had been confirmed. Thus, the toxicity of AuNP-S-mPEG itself was suggested to be negligible.

In C6 glioma cells, significant cytotoxicity was observed after in vitro treatment with ^211^At-AuNP-S-mPEG with a diameter of 120 nm labeled with 1 MBq/mL of radioactivity. In the PANC-1 cells, the cytotoxicity of ^211^At-AuNP-S-mPEG was similar to that seen in the C6 glioma cells. Thus, the cell toxicity of ^211^At-AuNP-S-mPEG as a non-targeted agent may not depend on the cell type.

The degree of cellular internalization of the 120 nm AuNP-S-mPEG as assessed by qualitative observation after 24 h of incubation (Fig. 2G) and that of ^211^At-AuNP-S-mPEG as assessed by quantitative evaluation after 3 h of incubation (Additional file 1: Fig. S4) were shown to be very high. For smaller AuNP-S-mPEG, however, higher concentrations were required for cellular internalization (Fig. 2B, D, F). Although the mechanism responsible for cellular internalization remains unknown, the 120 nm ^211^At-AuNP-S-mPEG might have precipitated to the bottom of the well used in the presently reported in vitro system (Additional file 1: Fig. S3), creating an extremely elevated concentration around cells that had adhered to the bottom of the culture. As a result, frequent cellular contact caused by close proximity with a high concentration of AuNP-S-mPEG might be involved in cellular internalization. ^211^At-AuNP-S-mPEG has not been modified to increase cell membrane permeability or to adhere to the cell membrane. Therefore, ^211^At-AuNP-S-mPEG is considered to be internalized via passive endocytosis depending on the concentration difference between the inside and the outside of the cell membrane.

Strong cytotoxicity was only confirmed (Fig. 1D) under conditions where internalization was observed (Fig. 2G, Additional file 1: Fig S4). Small AuNP-S-mPEG did not precipitate and would not have been internalized unless their concentration was greatly increased (Fig. 2B, D, F). Therefore, in vitro, no obvious cytotoxicity was observed at lower concentrations for small AuNP (Additional file 1: Table S3). These facts suggest that the cytotoxicity of ^211^At-AuNP-S-mPEG is caused by α irradiation, which is enhanced by the cellular internalization of the nanoparticles. When injected into cancer tissue, the ^211^At-AuNP-S-mPEG are thought to be first distributed in the intercellular fluid [24]. Intercellular fluid accounts for about 15% of the whole tissue, and the diffusion capability of nanoparticles is lower than that of a membrane-permeable solvent. Therefore, at the time of injection, the ^211^At-AuNP-S-mPEG is probably spatially concentrated around the tumor cells. The prolonged contact of cells in tissues with a high concentration of ^211^At-AuNP-S-mPEG may cause the nanoparticles to be internalized, thereby suppressing tumor cell growth.

Observations of the intratumoral distribution using autoradiography revealed that the 30 nm ^211^At-AuNP-S-mPEG had a high diffusivity in the tumor tissue, while the 120 nm ^211^At-AuNP-S-mPEG had a relatively low diffusivity. These observations suggest that the smaller the nanoparticle size, the greater the diffusivity in the tumor tissue. The tumor growth suppression effect of the α-rays from ^211^At-AuNP-S-mPEG was proportional to the size of the particles under the conditions of this study. Thus, for particles that are a minimum of 5 nm in diameter or larger, smaller ^211^At-AuNP-S-mPEG are considered to have greater diffusivity in tumor tissues and hence a greater tumor growth suppression effect. Unlabeled AuNP-S-mPEG did not show obvious cytotoxicity in either in vivo or in vitro studies. Thus, the antitumor property of the nanoparticles is clearly due to the cytotoxic effect of α-rays. ^211^At-AuNP-S-mPEG with diameters ranging between 5 and 120 nm did not accumulate systemically in any organs for at least 42 h, i.e. 6 half-lives of ^211^At after intratumoral administration. These results show that ^211^At-AuNP-S-mPEG does not undergo back-diffusion from the tumor tissue into blood vessels. On the other hand, as a result of the intratumoral administration of ^211^At-NaAt, almost all the ^211^At diffused from the tumor into the stomach and thyroid gland within 19 h of administration (Additional file 1: Fig. S5). These facts indicate that ^211^At does not dissociate from intratumorally administered ^211^At-AuNP-S-mPEG.

AuNP with a diameter of 10 nm or more reportedly exhibit vascular permeability and can be dispersed to various organs in a size-dependent manner after intravenous or gastrointestinal administration [25–27]. These inconsistencies with previous studies indicate that the vascular permeability of AuNP may depend on particle modification.

The effectiveness of targeting a specific protein for the purpose of nanoseed brachytherapy is controversial [15, 28]. In this study, even non-targeted nanoseeds were shown to have a tumor growth inhibitory effect and to exert cytotoxicity in two different types of tumor cells both in vitro and in vivo. In principle, this method is likely to be independent of the histological type of the malignant tumor and could therefore be easily used as a brachytherapy for many tumor types. The labeling of ^211^At-AuNP-S-mPEG is completed by a simple operation involving the stirring of AuNP-S-mPEG and ^211^At together. Therefore, the nanoparticles can be generated immediately before administration, allowing them to be labeled with a high specific radioactivity. In the present study, ^211^At-AuNP-S-mPEG showed a high and exclusive retention at the injected site, where ^211^At radioactivity was detected. These findings and the properties of α-rays suggest that very high-dose treatments with simple nanoparticles ^211^At-AuNP-S-mPEG might be feasible on an outpatient basis. In addition, Au and PEG are already clinically used as medicines, and their low toxicity has already been recognized [29, 30].

The present study had the following limitations. First, the mass concentration, particle concentration, and osmotic pressure of the administered solutions were difficult to control because the administration of a constant radioactivity concentration was prioritized. Second, since the radioactivity could not be further increased due to restrictions on the supply of ^211^At, the radioactivity level administered to the tumor was set at around 1.4 MBq. In a study examining the intratumoral administration of Lu-177-labeled AuNP-S-mPEG, however, a much stronger tumor growth suppression was observed at an administration level of 4.5 MBq, compared with 3.0 MBq administration [28]. Further study is thus necessary to optimize the dose in experiments using higher doses.

## Conclusions

The intratumoral single administration of ^211^At-AuNP-S-mPEG was capable of strongly suppressing the tumor growth of C6 glioma and PANC-1 cells. ^211^At-AuNP-S-mPEG ranging in diameter from 5 to 30 nm were shown to diffuse well locally after intratumoral administration without spreading to other organs throughout the body for at least 6 half-lives of ^211^At. A smaller particle size was thought to be more effective for suppressing tumor growth. Locally administered nanoseed brachytherapy using ^211^At-AuNP-S-mPEG was considered to be an effective and safe non-targeted anticancer treatment.

## Supplementary Information

**Additional file 1.** Additional text, figures, and tables.

## Data Availability

All data generated or analyzed during this study are included in this published article.

## Abbreviations

EBRT: External beam radiation therapy
LET: Linear energy transfer
AuNP: Gold nanoparticles
MEM: Modified Eagle medium
FBS: Fetal bovine serum
mPEG: Methoxypolyethylene glycol
PEG: Poly ethylene glycol
CCK8: Cell Counting Kit 8
