# Supplementary material for: Intratumoral administration of astatine-211-labeled gold nanoparticle for alpha therapy
Source: J Nanobiotechnology. 2021 Jul 28;19:223. doi: 10.1186/s12951-021-00963-9 (PMC8317303; doi:10.1186/s12951-021-00963-9)
Supplement: Supplementary file 1 — Additional file 1. Additional text, figures, and tables. [file 12951_2021_963_MOESM1_ESM.docx]

**Supplementary Information**

**Intratumoral administration of astatine-211-labeled gold nanoparticle for alpha therapy**

**Hiroki Kato^1*^, Xuhao Huang^2^, Yuichiro Kadonaga^3^, Daisuke Katayama^1^, Kazuhiro Ooe^1^, Atsushi Shimoyama^2^, Kazuya Kabayama^2^, Atsushi Toyoshima^3^, Atsushi Shinohara^2,3^, Jun Hatazawa^4^, Koichi Fukase^2,3^**

1. Department of Nuclear Medicine and Tracer Kinetics, Osaka University Graduate School of Medicine

2. Department of Chemistry, Graduate School of Science, Osaka University

3. Division of Science, Institute for Radiation Sciences, Osaka University

4. Research Center for Nuclear Physics, Osaka University

* Corresponding author: Hiroki Kato, kato-h@umin.org

Department of Nuclear Medicine and Tracer Kinetics, Osaka University Graduate School of Medicine, 2-2 Yamadaoka Suita, Osaka 565-0871 Japan,

TEL: +81-6-6879-3461, FAX: +81-6-6879-3469

Compound syntheses

*Production and separation of ^211^At*

^211^At was supplied from RIKEN via Supply Platform of Short-lived Radioisotopes. ^211^At was produced in the ^209^Bi(α, 2n)^211^At nuclear reaction using the AVF Cyclotron. The metallic Bi target was prepared by a vacuum evaporation method onto an Al plate. The separation of ^211^At from the irradiated Bi target was conducted by a dry distillation method. The irradiated Bi target was placed in a quartz column and heated up to 850 °C using an electric tubular furnace. The evaporated ^211^At was transported from the quartz column by a carrier gas (mixed nitrogen and oxygen gas) into a Teflon trap tube which was cooled by ice water. The trapped ^211^At was then washed out by 100 μL of distilled water from the trap tube and received by a Teflon sample tube. The radioactivity of ^211^At dissolved in distilled water was determined by measurement of X-ray from a daughter nuclide, ^211^Po, using a high-purity germanium (HPGe) detector (BE-2020; Mirion Technologies (Canberra), Inc., Connecticut, USA).

*Synthesis of AuNP*

5 nm gold nanoparticles were purchased from Sigma-Aldrich, Co., LLC (St. Louis, USA), and 13 nm, 30 nm, and 120 nm gold nanoparticles were synthesized using the seeded growth synthesis method (Fig. S1A).[^1^](#_ENREF_1)

Tetrachloroauric (III) acid tetrahydrate was purchased from Kishida Chemical Co., Ltd. (Osaka, Japan) and used as a raw material for gold nanoparticles synthesis.

The following aqueous solutions (A-D) were prepared: A) 8 mL of water was added to 2 mL of tetrachloroauric (III) acid (0.17% w/v) aqueous solution for adjustment; B) 0.5 mL of ascorbic acid (1% w/v) aqueous solution and 0.25 mL of sodium citrate (0.88% w/v) aqueous solution were mixed, and 9.25 mL of water was added for adjustment; C) 2 mL of water was added to 8 mL of tetrachloroauric (III) acid (0.17% w/v) aqueous solution for adjustment; and D) 2 mL of ascorbic acid (1% w/v) aqueous solution and 1 mL of sodium citrate (0.88% w/v) aqueous solution were mixed, and 7 mL of water was added for adjustment.

Next, 47.5 mL of water was added to 2.5 mL of a tetrachloroauric (III) acid (0.17% w/v) aqueous solution. After the addition was completed, the temperature was raised to 100 °C or higher with stirring. After heating, 2 mL of an aqueous solution of sodium citrate (0.88% w/v) and citric acid (0.05% w/v) were add to the aqueous solution and the solution was stirred for 5 minutes at the same temperature. After stirring, the temperature was returned to room temperature to obtain 13 nm gold nanoparticles (AuNP).

Next, 15 mL of water was added to 5 mL of 13 nm AuNP solution for adjustment. While stirring this solution, aqueous solutions A and B were simultaneously added from separate syringes at a flow rate of 0.25 mL/min. After the addition was completed, the temperature was raised to 100 °C or higher with stirring, and the mixture was further stirred for 30 minutes at the same temperature. After stirring, the temperature was returned to room temperature to obtain 30 nm AuNP.

Finally, 15 mL of water was added to 5 mL of 30 nm AuNP solution for adjustment. While stirring the prepared aqueous solution, aqueous solutions A and B were simultaneously added from separate syringes at a flow rate of 0.25 mL/min. After the addition was completed, the temperature was raised to 100 °C or higher with stirring, and the mixture was further stirred for 30 minutes at the same temperature. After stirring, the temperature was returned to room temperature to obtain 60 nm AuNP. While stirring 20 mL of the 60 nm AuNP solution, aqueous solutions C and D were simultaneously added from different syringes at a flow rate of 0.25 mL/min. After the addition was completed, the temperature was raised to 100 °C or higher with stirring, and the mixture was further stirred for 30 minutes at the same temperature. After stirring, the temperature was returned to room temperature to obtain 120 nm AuNP.

*Modification of AuNP with mPEG*

All sizes of AuNPs were modified with methoxy poly(ethylene glycol) thiol (Fig. S1A). To modify the AuNP, poly (ethylene glycol) (PEG) methyl ether (mPEG) thiol (Mn 6,000) purchased from Sigma-Aldrich, Co., LLC (St. Louis, USA) was used. mPEG thiol (Mn 6,000) was added to the aqueous solutions containing the 5 nm, 13 nm, 30 nm, and 120 nm AuNP so that the final concentration of mPEG thiol was 0.1 mg/mL. The reaction mixtures were stirred at room temperature for 2 hours. After that, the mPEG-modified AuNP (5 nm) was centrifuged by using amicon ultra-0.5 centrifugal filter devices (50K) at 10000 G for 10 minutes. After centrifugation, distilled water was added to the devices. This operation was performed 3 times to obtain 5 nm AuNP-S-mPEG. The mPEG-modified AuNP (13 nm, 30 nm, 120 nm) was precipitated by centrifugation (10000 G, 1 hour). After removing the supernatant liquid by decantation, the same amount of distilled water as the removed solution was added to the precipitate. This operation was performed twice to obtain 13, 30, and 120 nm AuNP-S-mPEG. The quality of the synthesized AuNP-S-mPEGs were confirmed using transmission electron microscopy (TEM) (JEM-2100; JEOL Ltd., Tokyo, Japan). The sizes of the nanoparticles were measured from the TEM images using the software ImageJ (https://imagej.nih.gov/ij/).

*^211^At labeling on AuNP-S-mPEG*

^211^At aqueous solution was added to AuNP-S-mPEG aqueous solution and the resulting mixture was shaken at room temperature for 15 minutes. After shaking, an ^211^At-AuNP-S-mPEG aqueous solution (about 40 MBq/mL) was obtained (Fig. S1A). The ^211^At-AuNP-S-mPEG aqueous solution was diluted with physiological saline before administration to rats. The radioactivity of ^211^At-AuNP-S-mPEG was measured using a germanium semiconductor detector. The yield was evaluated using the centrifugal filter separation method. Briefly, the reaction solution was added to the amicon ultra-0.5 centrifugal filter devices, then centrifuged to separate the ^211^At-AuNP-S-mPEG and free ^211^At. After the first centrifugation, water was added to the filter device then second and third centrifugation were performed to obtain the filtrates. The radioactivity of the filtrates and the ^211^At-AuNP-S-mPEG solution in the filter devices were measured to evaluate the reaction yield.

Compound characterizations

Multi-angle dynamic light scattering (MADLS) and zeta potential measurements of AuNP (Table S1) and AuNP-S-mPEG (Table S2) were made using Zetasizer Ultra (Malvern Panalytical Ltd., Malvern, UK). Each sample was added to the specified cell, and the MADLS mode or zeta potential mode was then chosen to measure the hydrodynamic diameter and zeta potential, respectively; each of the measurements was made in triplicate. The hydrodynamic diameter of AuNP-S-mPEG remained stable over time and was larger than that of AuNP for each nanoparticle size. This result shows the stability of mPEG modification of the gold nanoparticles.

The mean core diameters of 5-nm, 13-nm, 30-nm and 120-nm AuNP-S-mPEG were also measured prior to administration in vitro or in vivo by transmission electron microscopy (TEM), and were found to be 5.1 ± 1.1 nm, 13.1 ± 1.4 nm, 30.8 ± 2.7 nm and 120.7 ± 13.3 nm, respectively (Table S3, S4). Using negatively stained TEM images, the size distributions were measured using ImageJ (Fig. S1C, E, G, I); the results revealed that the AuNP-S-mPEG particles were approximately spherical and reasonably monodispersed. The coated mPEG molecules were confirmed as a white and blurred signal around AuNP (Fig. S1B, D, F, H).

AuNP-S-mPEG remained stable after incubation in saline for 24 hours, with no apparent aggregation (Fig. S1J-M).

The mass concentrations of Au in the 5 nm, 13 nm, 30 nm, and 120 nm AuNP-S-mPEG solutions were measured using ICP-OES (Optima 8300; Perkin Elmer Inc., Waltham, USA) and were found to be 63.6 mg/L, 42.1 mg/L, 43.3 mg/L, and 168 mg/L, respectively.

The mass concentrations of AuNP-S-mPEG for the cell toxicity study and the intratumoral administration are shown in the Table S3 and S4 respectively. The particle concentrations, which were not directly measured but were calculated from the mass concentration, and the radiation dose administered to each tumor are shown in the table S4.

References

**1.** Ziegler C, Eychmüller A. Seeded Growth Synthesis of Uniform Gold Nanoparticles with Diameters of 15−300 nm. *The Journal of Physical Chemistry C.* 2011;115:4502-4506.

Supplemental table

Table S1.

Hydrodynamic diameter and Zeta-potential of AuNP

|  | Hydrodynamic diameter in citrate solution  / nm | Zeta-potential in citrate solution  / mV |
| --- | --- | --- |
| 5 nm AuNP ^a^ | 11.9 ± 0.1 | -14.7 ± 1.7 |
| 13 nm AuNP | 13.9 ± 0.5 | -43.3 ± 3.1 |
| 30 nm AuNP | 29.8 ± 0.7 | -35.9 ± 0.3 |
| 120 nm AuNP | 120.4 ± 5.4 | -40.5 ± 0.8 |

*^a^*5-nm AuNP was purchased from Sigma-Aldrich and was supplied in citrate buffer and a proprietary stabilizing solution.

Table S2.

Hydrodynamic diameter and Zeta-potential of AuNP-S-mPEG

|  | Hydrodynamic diameter  in water / nm | Hydrodynamic diameter  in saline (3 h) ^a^ / nm | Hydrodynamic diameter  in saline (24 h) ^b^ / nm | Zeta-potential  in water / mV |
| --- | --- | --- | --- | --- |
| 5 nm  AuNP-S-mPEG | 22.2 ± 0.9 | 23.7 ± 1.5 | 27.7 ± 1.0 | -40.1 ± 1.6 |
| 13 nm  AuNP-S-mPEG | 35.9 ± 0.3 | 37.3 ± 1.0 | 38.3 ± 1.0 | -17.9 ± 0.6 |
| 30 nm  AuNP-S-mPEG | 49.2 ± 0.3 | 50.5 ± 1.9 | 48.6 ± 2.4 | -21.0 ± 1.6 |
| 120 nm  AuNP-S-mPEG | 138.9 ± 0.7 | 142.6 ± 1.6 | 147.4 ± 0.7 | -44.9 ± 0.6 |

*^a^*After the incubation of AuNP-S-mPEG in saline for 3 h at room temperature, the hydrodynamic diameter was measured.

*^b^*After the incubation of AuNP-S-mPEG in saline for 24 h at room temperature, the hydrodynamic diameter was measured.

Table S3.

Gold nanoparticles administered to cultured cells for the cell toxicity study

| Particles | Diameter (nm) | Mass concentration (mg/L) | | | |
| --- | --- | --- | --- | --- | --- |
|  |  | 0 MBq/mL group | 0.01 MBq/mL group | 0.1 MBq/mL group | 1 MBq/mL group |
| 5 nm | 5.1 (1.1) | 11.2 | 0.11 | 1.12 | 11.2 |
| 13 nm | 13.1 (1.4) | 6.07 | 0.06 | 0.61 | 6.07 |
| 30 nm | 30.8 (2.7) | 7.62 | 0.08 | 0.76 | 7.62 |
| 120 nm | 120.7 (13.3) | 29.7 | 0.30 | 2.97 | 29.7 |

Mass concentration: The mass concentration of Au of the particles in the solvent.

The standard deviations are shown in parentheses.

Table S4.

Gold nanoparticles administered to each tumor

| Animal model | Particles | Diameter (nm) | Particle concentration (/L) | Mass concentration (mg/L) | Dose (MBq) |
| --- | --- | --- | --- | --- | --- |
| C6 | 5 nm | 5.1(1.1) | 9.46 × 10^15^ (1.65 × 10^15^) | 12.7 (2.21) | 1.39 (0.04) |
|  | 13 nm | 13.1 (1.4) | 2.46 × 10^14^(4.92 × 10^13^) | 5.59 (1.12) | 1.46 (0.07) |
|  | 30 nm | 30.8 (2.7) | 2.95× 10^13^(2.76 × 10^13^) | 8.74 (8.18) | 1.29 (0.50) |
|  | 120 nm | 120.7 (13.3) | 2.38 × 10^12^(2.53 × 10^12^) | 42.4 (45.0) | 1.51 (0.57) |
|  | Unlabeled 30 nm | 30.8 (2.7) | 6.49 × 10^13^ | 19.2 | 0 |
| PANC-1 | 13 nm | 13.1 (1.4) | 1.15 × 10^15^ (1.94 × 10^14^) | 26.2 (4.40) | 1.17 (0.07) |
|  | Unlabeled 13 nm | 13.1 (1.4) | 1.23 × 10^15^ (2.13 × 10^14^) | 27.8 (4.83) | 0 |

Mass concentration: The mass concentration of Au of the particles in the solvent.

The standard deviations are shown in parentheses.

Supplemental Figures


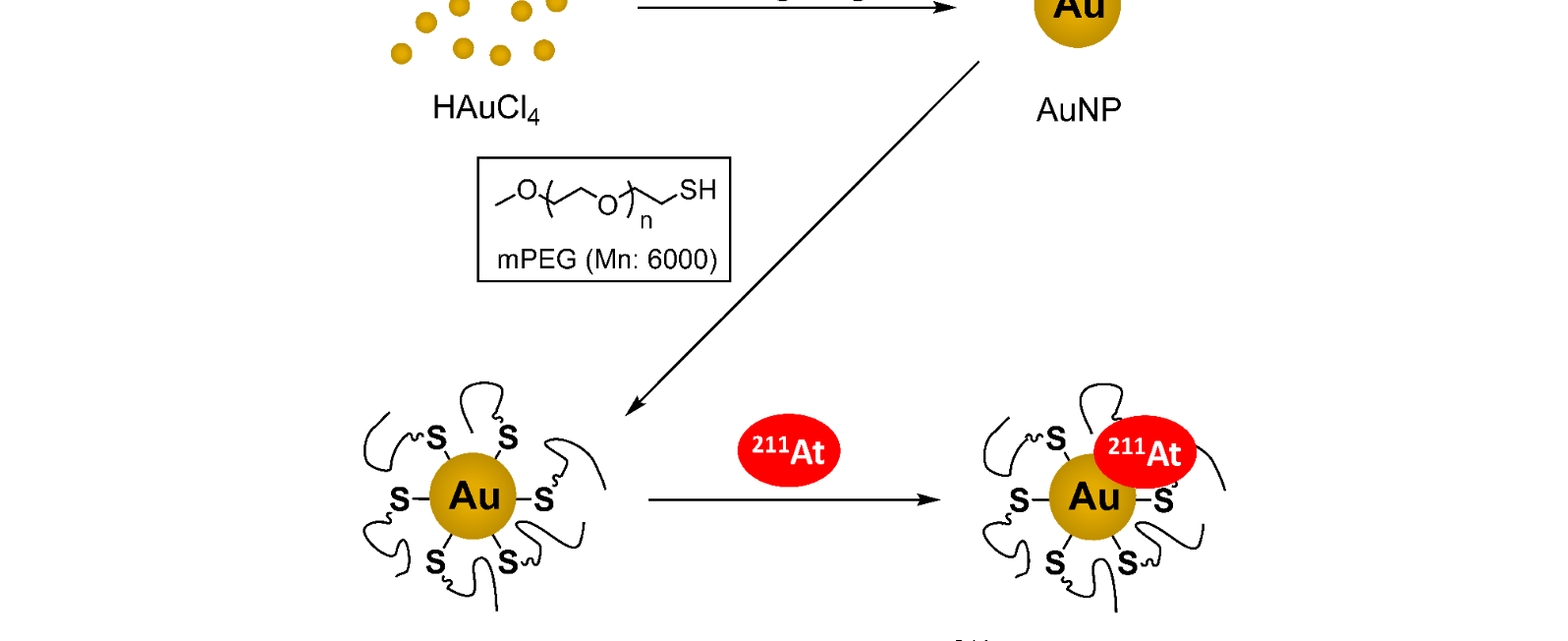
Fig. S1


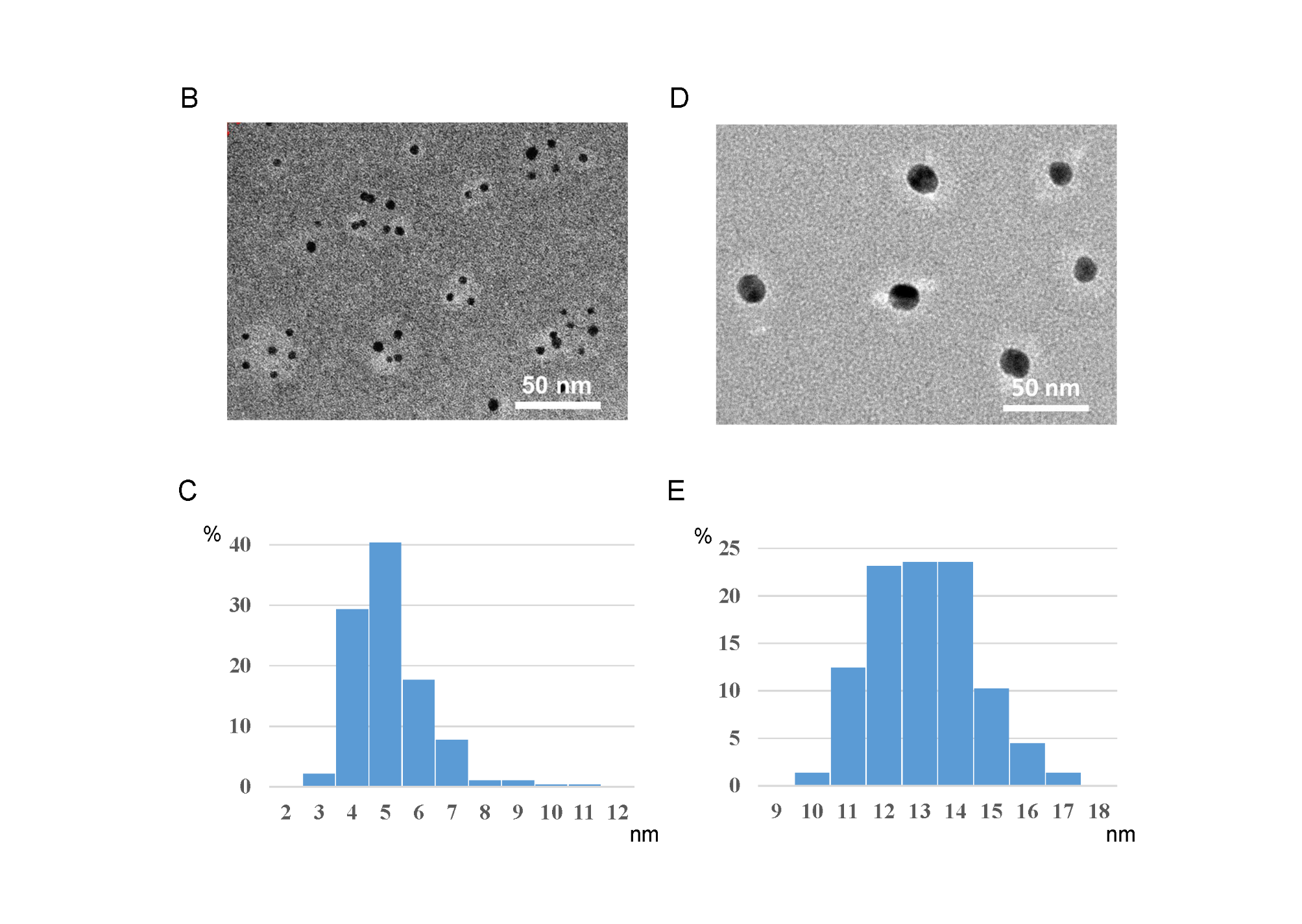


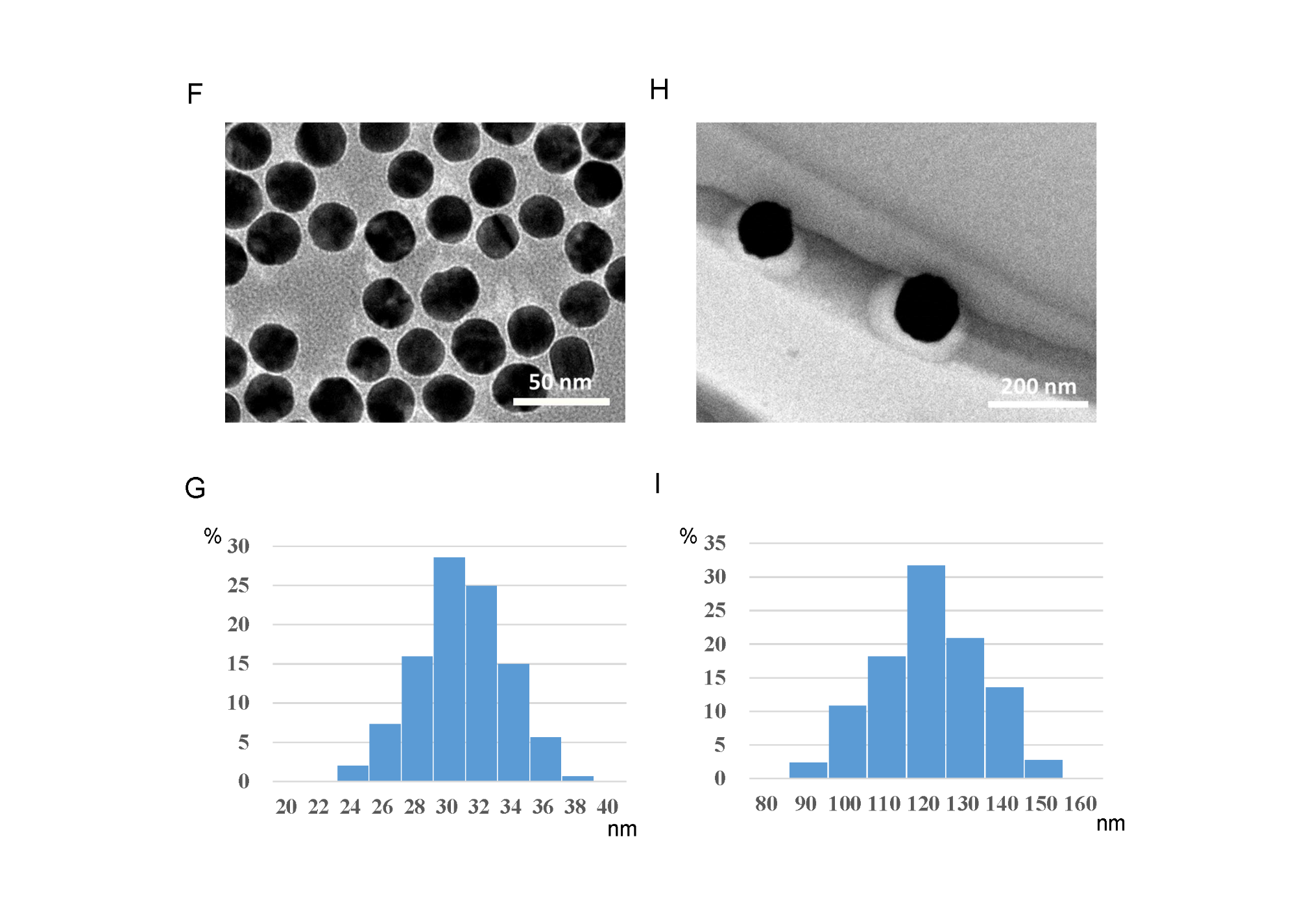


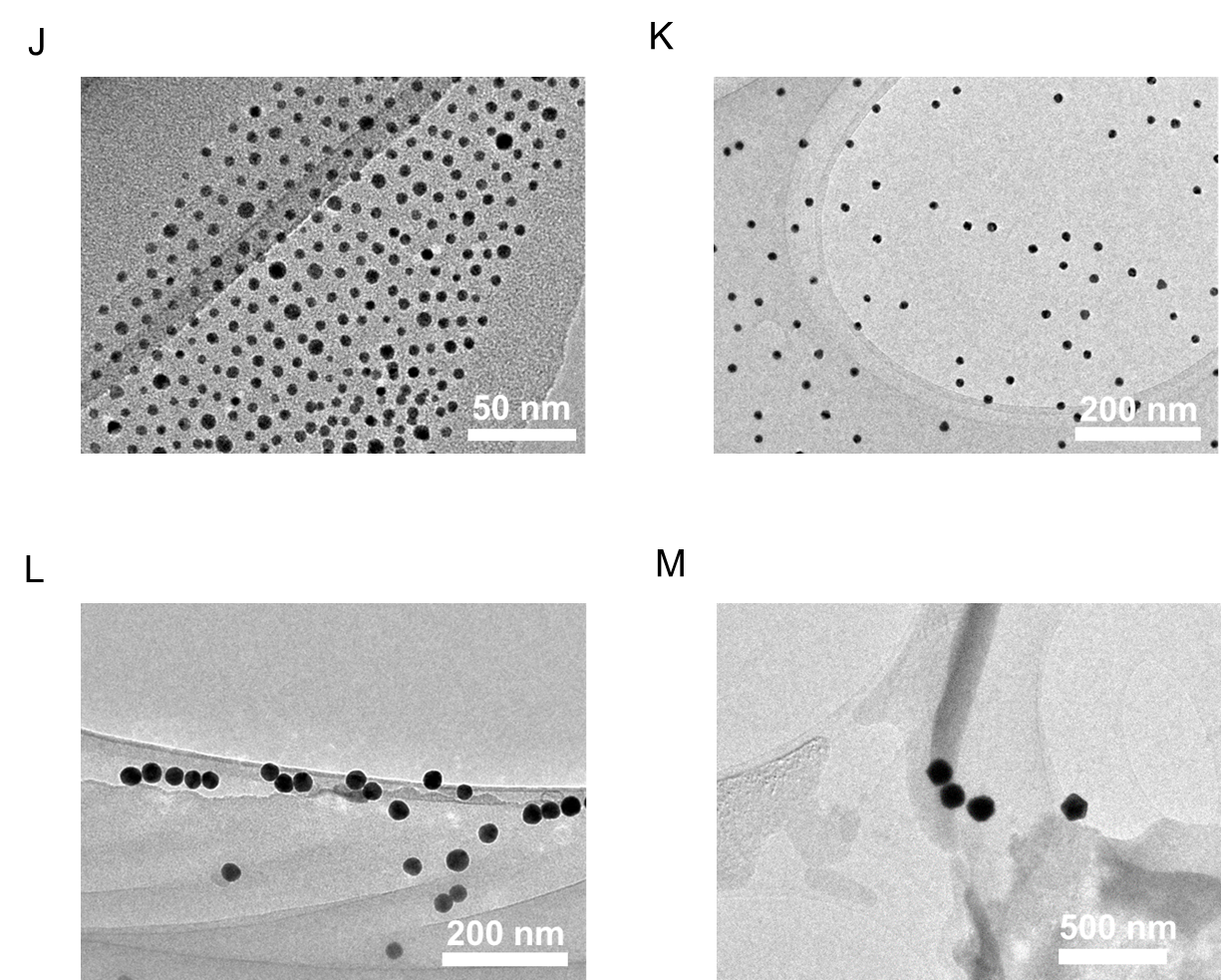


Fig. S1.

(A)

AuNP nanoparticles of the desired size were produced from tetrachloroauric (III) acid; mPEG thiol was added, and the mixture was stirred to produce AuNP-S-mPEG. ^211^At-AuNP-S-mPEG was then produced by adding ^211^At aqueous solution to the AuNP-S-mPEG aqueous solution and shaking.

(B, D, F, H)

Negatively stained TEM images showing mPEG-coated gold nanoparticles with diameters of 5 nm (B), 13 nm (D), 30 nm (F), and 120 nm (H). The white blur around the gold nanoparticles is the coated mPEG molecule.

(C, E, G, I)

Size distributions of 5 nm (C), 13 nm (E), 30 nm (G), and 120 nm (I) mPEG-coated gold nanoparticle cores. The sizes of the nanoparticle were measured using TEM images and ImageJ.

(J, K, L, M)

TEM images of 5-nm (J), 13-nm (K), 30-nm (L), and 120-nm (M) mPEG-coated gold nanoparticle cores after incubation in saline for 24 hours.


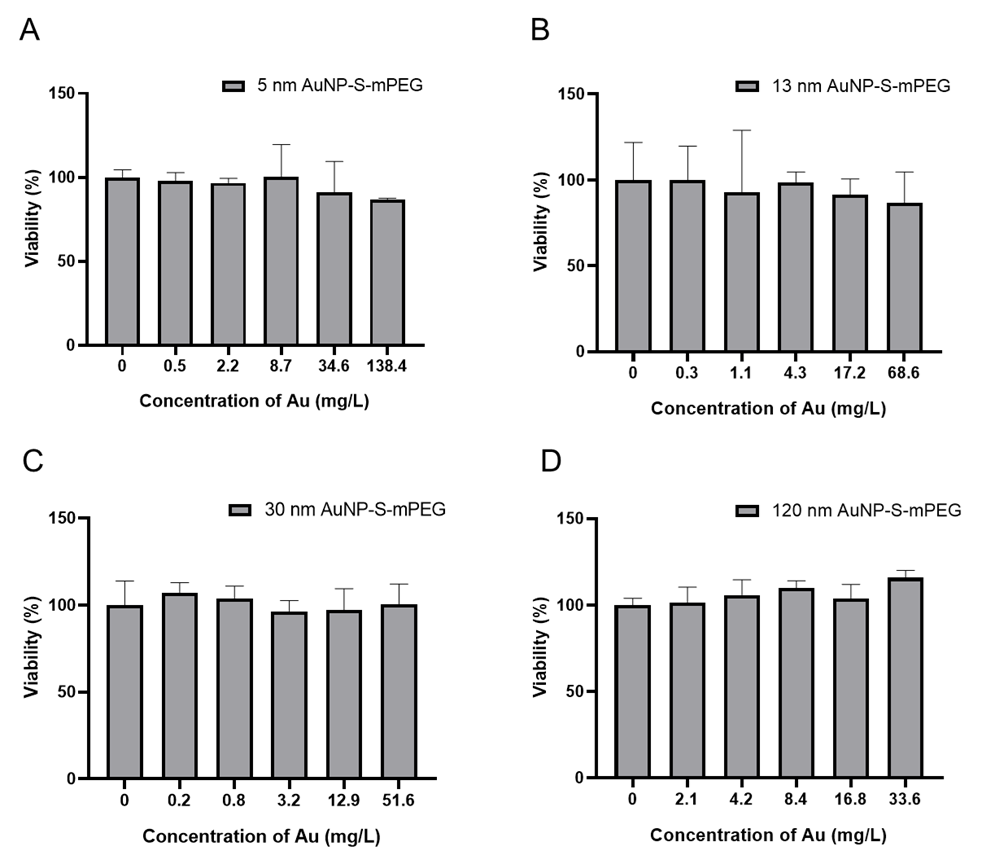
Fig. S2.

Fig. S2.

C6 glioma cells were incubated with unlabeled AuNP-S-mPEG with a diameter of 5 nm (A), 13 nm (B), 30 nm (C) or 120 nm (D) at different concentrations for 24 hours. For the evaluation of cell viability, CCK8 Kit was used. WTS-8 solution was added, then the cells were cultured for 2 hours. Viability was calculated as described in the legend of Fig.1

Fig. S3.


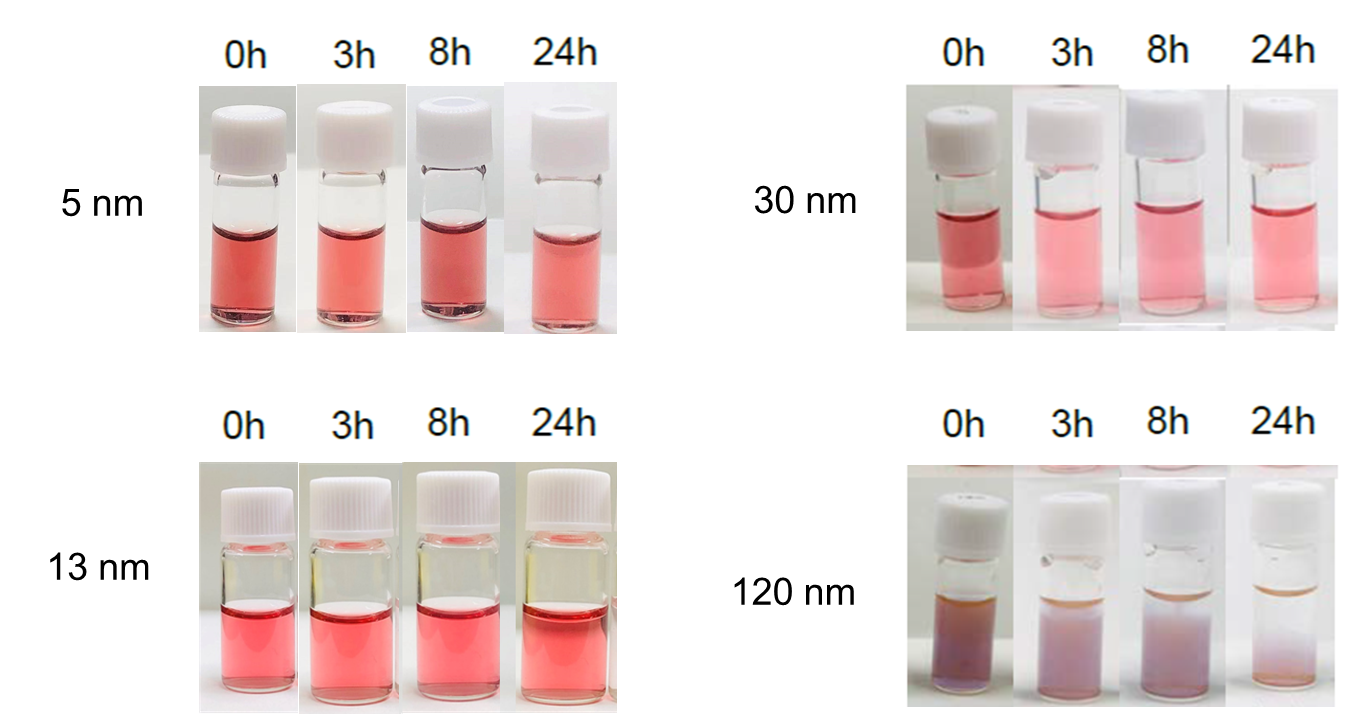


Fig. S3.

AuNP-S-mPEG aqueous solutions containing each nanoparticle size (5 nm, 13 nm, 30 nm and 120 nm) were allowed to stand and were then observed 24 hours later. The 120 nm AuNP-S-mPEG had precipitated after 24 hours, and the concentration at the bottom of the container had increased.

Fig. S4.

Fig. S4.

To evaluate the uptake of ^211^At-AuNP-S-PEG and ^211^At solution by C6 glioma cells, the cells (2 × 10^4^ cells/well in 100 μL medium) were seeded on to 96-well plates and the different kinds of solutions were added to each well. The Au concentration in each group (i.e., 5 nm, 13 nm, 30 nm, or 120 nm) was the same as that in the 1 MBq/mL group in the in vitro cell toxicity study (Table S3). Each sample was added to the cells and incubated for 3 hours. After incubation, the medium was removed, and cells were washed twice with 1X PBS, and lysed with 1N NaOH. The radioactivity levels in the lysed cell samples were measured with the gamma counter, 2480 Wizard^2^ (PerkinElmer, Inc.). The protein levels were measured with a BCA protein assay kit (Thermo Scientific).

Each group was compared with the control group (n = 3).

****p* < 0.001.

CPM: Count per minute

Fig. S5.


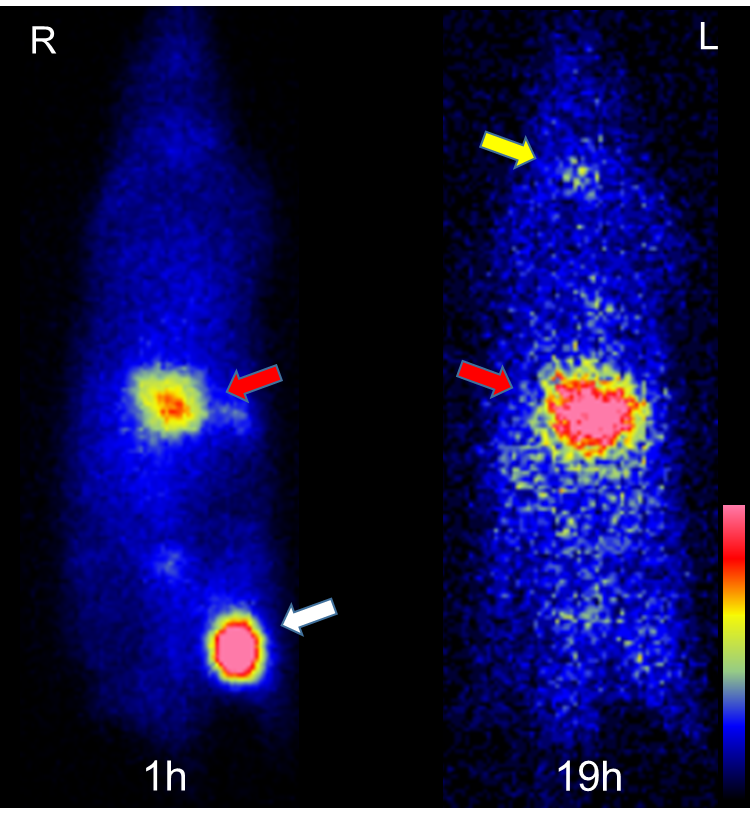


Fig S5.

C6 glioma cells were subcutaneously transplanted into the left lower extremity of an 8-week-old male nude rat in the same manner as for the other rats described in the text.

Next, 0.0l mL of ^211^At solution was added to 1 mL of sodium bicarbonate and ascorbic acid mixing solution (1.0% ascorbic acid, 2.3% NaHCO_3_), and the solution was allowed to stand for 1 hour at room temperature to obtain an ^211^At-NaAt solution. Then, 1 mL of ^211^At-NaAt solution with a radioactivity concentration of 4.74 MBq/mL, adjusted with saline, was injected into the tumor at 8 weeks after transplantation. Scintigraphy was performed at 1 and 19 hours after administration. Almost all the radioactivity in the tumor (white arrow) diffused and eventually accumulated in the stomach (red arrow) and thyroid gland (yellow arrow) at 19 hours after administration.
